# Recuperative Power

**Published:** 1864-02

**Authors:** 


### RECUPERATIVE POWER.

THIS is the "vis medicatrix naturæ," the power of nature to cure herself, and is implanted by Divinity in all that lives, vegetable or animal. Were there no such power, every injury done to blade of grass, or shrub, or tree, to insect, animal, or man, would result in sickness, decay, and death; and soon there would not be a living thing the globe over.

The extent to which injuries to the human frame may be recovered from are sometimes amazing; nature only asking one thing, and that is, to be let alone; to be allowed quietude, rest. Soon after breakfast, March sixteenth, 1860, Mrs. L. A. Page, two months married, was captured in the Rocky Mountains by a band of Apache Indians. Although she had suffered much from recent attacks of fever and ague, she traveled all day on foot, over a rough, mountainous road; to increase her pace, they frequently pointed their six-shooters at her head. At the end of sixteen miles, she lagged so much behind that her captors resolved to kill her, and for that purpose removed every part of her clothing except a single garment; they next threw their lances at her, inflicting eleven wounds in her body, and threw her over a rocky precipice, and then threw stones at her, several of which struck her on the head; this was about sunset. Having alighted on a bank of snow, the fall was broken; still she was insensible, and must have remained there, in that condition, for two nights and a day. That she should have survived an hour, is extraordinary. When she recovered her senses, she put snow on her wounds, and started in the supposed direction of her home. Her feet gave out the first day, and she was compelled to crawl. Sometimes, after crawling up a steep ledge of the mountain, laboring hard for half a day to accomplish it, she would lose her footing and slide down to a lower part than that from which she started. At night she scratched holes in the sand in which to sleep, in order to protect herself from the cold winds of March on the mountain, five thousand feet above the level of the sea; but before she could start on her daily travels, she had to remain until the sun warmed her up; having no fire, and not a particle of clothing beyond the inner garment. She lived on grass—nothing else. On the fourteenth day she reached an untenanted camp of workmen in the Pineries, where she found a little food, and some flour which had been spilled on the ground. The fire being not quite out, she kindled it up, and made a little cake of the flour which she had scraped up, being the first food she had tasted since the morning she left home. She could hear the men at work, and sometimes see them, but could not attract their attention. She, however, made out to crawl to a path which she knew they would have to pass at night, whence they carried her to her home, two miles distant; after an absence of sixteen days, she was well again.

This narration is given to impress on the mind of the reader that nature is the best physician, as a general rule; that her requirements are few and simple, and that with pure air, pure water, and quietude, ailments may be recovered from which, had they been removed by any human remedy, would have given the discoverer a world-wide name, and have poured into his treasury untold millions. The first remedies in this case were perfect quietude for two days; thus, every particle of strength preserved by the system was applied toward repairing the injury done. All this while, however, the purest air was breathed, every breath of which, while it imparted life to the blood, left the body loaded with its impurities of waste and decay, and particles dead either by disease or the physical violences offered. Next to this rest and pure air, was either snow-water, or that from mountain-springs, according as it could be obtained

Another important element in nature's cure of violence and sickness is in dieting. In almost all cases of ordinary disease, nature, in self-defense, takes away the appetite, so that what power the system has should be employed in throwing out diseased matters and those which oppress it, instead of expending that power in the digestion of food, which would only serve to clog the almost-stopped machinery.

For two weeks she ate nothing but grass. A most curious and valuable practical truth is taught here, one worth millions in the cure of disease, and of a world-wide application; a truth which daily forces itself on every intelligent physician and every observant nurse, that the diet of the sick should be simple, single, unconcentrated; that is, one thing at a time, and that should be an article which contains but a little nutriment in a large amount of gross. Hence, in all ordinary sickness, acute diseases, such as fevers, fruits and vegetables are better than meat; for while the latter is nearly all nutriment, nine tenths of the former are waste. In the common apple or turnip there are, perhaps, five atoms of nourishment in a hundred atoms of gross. There is still a less proportion of nutriment in grass, but there is some, and there is power in the human stomach to extract it. There is one item in connection with the sick which is of untold value; more and better and purer nutriment is obtained by a sick man's stomach from a smaller amount of food than from a larger; from one ounce, than a dozen. The single ounce may be thoroughly digested, hence all the blood it makes is perfectly pure, is full of strength and life; while the pound, being more than the stomach can take care of, is imperfectly digested, and must make an imperfect blood material; hence can impart no radical, enduring strength. But this is not all; this imperfect blood is mixed as soon as made with the whole blood of the body, and to that extent renders the entire mass impure. The lesson of this article is this: In all ordinary ailments and accidents, secure quiet of body, composure of mind, pure air, pure water, and simple food at regular intervals, being a little hungry all the time.

